# The Catsper channel and its roles in male fertility: a systematic review

**DOI:** 10.1186/s12958-017-0281-2

**Published:** 2017-08-15

**Authors:** Xiang-hong Sun, Ying-ying Zhu, Lin Wang, Hong-ling Liu, Yong Ling, Zong-li Li, Li-bo Sun

**Affiliations:** 1 Department of pharmacy, the affiliated hospital of Qingdao University Medical College, Qingdao, 266555 China; 2 Department of pharmacy, College of pharmacy of Qingdao University, Qingdao, China; 3 Department of clinical laboratory, the affiliated hospital of Qingdao University Medical College, Qingdao, China

**Keywords:** Catsper channel, Ca^2+^ signal, Male infertility, Medicine

## Abstract

The Catsper channel is a sperm-specific, Ca^2+^-permeable, pH-dependent, and low voltage-dependent channel that is essential for the hyperactivity of sperm flagellum, chemotaxis towards the egg, capacitation and acrosome reaction. All of these physiological events require calcium entry into sperm cells. Remarkably, Catsper genes are exclusively expressed in the testis during spermatogenesis, and are sensitive to ion channel-induced pH change, such as NHEs, Ca^2+^ATPase, K^+^ channel, Hv1 channel and HCO_3_^−^ transporters. Furthermore, the Catsper channel is regulated by some physiological stimulants, such as progesterone, cyclic nucleotides (e.g., cAMP, cGMP), zona pellucida (ZP) glycoproteins and bovine serum albumin (BSA). All of these factors normally stimulate Ca^2+^ entry into sperm through the Catsper channel. In addition, the Catsper channel may be a potential target for male infertility treatment or contraception. This review will focus on the structure, functions, regulation mechanisms and medicinal targets of the Catsper channel.

## Background

Mature mammalian sperm execute many important physiological processes, such as sperm hyperactivation, chemotaxis towards the egg, capacitation and acrosome reaction, before even entering the female reproductive tract and contacting eggs for fertilization. Most studies show that all of physiological processes are closely related to the change of the calcium ion concentration ([Ca^2+^]i) [1, 2] in sperm. There are two main sources for Calcium ions in sperm: (1) some of them are stored in a calcium pump located in the head of sperm, a redundant nuclear envelope in the same position as the IP3 receptor in the neck region, and (2) others are packed in the mitochondria of the midpiece [3, 4]. Some processes in sperm depend on calcium ion channels opening in the cell membrane [5]. Several typical voltage-gated Ca^2+^ channels are located in testis, but most voltage-gated Ca^2+^ channels also take effect in other organs, such as brain and heart. It is only the Catsper ion channel that is exclusively expressed in spermatozoa. The whole-cell patch-clamp technique applied to mouse spermatozoa, showed direct electrophysiological characteristics of the protein channels. Ca^2+^ currents were only detected in the Catsper channel. Catsper is a sperm- specific, Ca^2+^-permeable, pH-sensitive and weakly voltage-dependent ion channel that is located in the membrane of the flagellar principal piece, The presence of an inactive Catsper protein in male mice induces infertility [6].

The Catsper channel is activated by intracellular alkaline pH, as shown by whole-cell patch-clamp in 2006 [5]. This channel not only permits Ca^2+^ entry into spermatozoa under physiological conditions but also allows monovalent cations (Na^+^, Cs^+^) or a divalent cation (Ba^2+^) to pass into spermatozoa if there is no extracellular Ca^2+^. The Catsper channel complex contains four α subunits (Catsper1–4 [5, 7]) and at least three auxiliary subunits (Catsper β (beta), Catsper γ (gamma) and Catsper δ (delta) [8]). The first pore-forming Catsper subunit, Catsper1, was discovered in 2001 [9] and plays a vital role in spermatozoa motility. Catsper1 was detected during a search for sequence homologies to voltage-gated Ca^2+^ selective channels. Previous studies showed that there is no Catsper1 expression in Catsper2-lacking mice and also no Catsper2 expression in Catsper1-lacking mice [7]. These results indicated that stable expression of Catsper1 requires Catsper2, and vice versa. However, Catsper3 and Catsper4 proteins are expressed in Catsper1-deletion mice, suggesting that stable expressions of Catsper3 and Catsper4 does not depend on the expression of Catsper1 and Catsper2 [10]. Compared with unselected sperm, a much higher proportion of swim-up selected sperm expresses Catsper1 [11], suggesting that Catsper1 also plays a crucial role in sperm swimming. Further studies showed that Catsper null sperm cells could not be hyperactivated under physiological conditions [1, 12]. Interestingly, depolarization evoked an increase in intracellular Ca^2+^ ([Ca^2+^]i) in WT sperm cells, but not in Catsper1 null spermatozoa [12]. The phenotypes of Catsper2^−/−^, Catsper3^−/−^ and Catsper4^−/−^ mice were indistinguishable from Catsper1^−/−^ mice, and their sperm also lacked the hyperactivate motility needed for fertilization [7, 13]. A whole sperm patch clamp of epididymal sperm showed that Catsper current is absent in Catsper1^−/−^, Catsper2^−/−^, Catsper3^−/−^ and Catsper4^−/−^ mice [6, 7].

In recent years, the study on Catsper channel activators and inhibitors is surging. Wang HF found that Cadmium (Cd), a heavy metal and endocrine disruptor in environment, caused male infertility through reducing the expression of Catsper proteins [14]. Mannowetz N also conducted a study that pregnenolone sulfate as a Catsper activator. However, pristimerin and lupeol which both are steroid-like molecules can act as contraceptive compounds through affecting sperm hyperactivation [15]. Therefore, the Catsper ion channel has been implicated as a potential target for male infertility treatment and contraception. Nonetheless, the role of Catsper and its antagonist in these functions have been scarcely reported. Herein, this review focuses on the role of Ca^2+^ signaling in sperm function and the effects of medicines targeting the Catsper channel.

## The structure and localization of the Catsper channel complex

The Catsper channel complex is encoded by at least seven genes. The structure and distribution of the Catsper subunits are all essential for the channel’s function [7]. Catsper1–3 are specifically expressed in the testis, while Catsper4 is predominantly expressed in the testis, and there is also weak expression in placenta and lung tissues [16]. The Catsper family is confined to the principal piece of mature spermatozoa flagella in humans and animals. There is no organelle in the principal piece of spermatozoa, so researchers speculate that Catsper1–4 are localized to the principal piece’s plasma membrane and involved in the regulation of flagella whipping.

Sequence homology of the four Catsper subunits in the transmembrane region is rare, and the variance ranges from 16 to 22% [5]. Catsper1 has a 21% homologous identity and 40% homologous similarity to Catsper2. Furthermore, the Catsper family also has relatively low sequence homologies among different species. The basic information of the seven Catsper genes in human and mouse are clearly shown in Table 1 and Table 2. The Catsper protein is widely expressed in mammals (mouse, rat, dog, human), sea squirt (*Ciona intestinalis*), and sea urchin (*Strongylocentrotus purpuratus*). Six transmembrane segments (S1-S6) are detected in separate pore-forming Catsper α subunits, forming two individual physiological active sites: the voltage sensor domain (S1-S4) and pore-forming domain (S1-P loop- S6). S1-S4 are linked by a short cyclic structure, and it is noteworthy that there are several positively charged amino acid residues (lysine/arginine) in the fourth transmembrane segment (S4) functioning as a voltage sensor [8]. S5 and S6 are linked by a short and hydrophobic cyclic structure, and this region has a conserved homologous sequence ([T] × [D] × [W]), which selectively permits Ca^2+^ entry through the cell membrane. Catsper1 contains six neutral amino acid residues in voltage-sensitive channels, while Catsper2 contains four such residues and Catsper3 and Catsper4 contain only two. Four αsubunits have coiled proteins in their C ends, forming a functional tetramer to constitute a whole channel. Catsper β, a recently discovered protein, contains two presumed transmembrane-spanning domains. Catsper β is also the first identified auxiliary protein of the Catsper channel, expressed predominantly in the testis and the sperm tail [17].

**Table 1:** The essential information of Catsper subunits in human testes

Gene name	Chromosome (human)	exon	Amino acid
Catsper1	11q13.1	12	780
Catsper2	15q15.3	14	530
Catsper3	5q31.1	8	344
Catsper4	1p36.11	11	472
Catsperβ	14q32.12	27	1116
Catsperγ	19q13.2	36	1159
Catsperδ	19p13.3	25	798

**Table 2:** The essential information of Catsper subunits in mice testes

Gene name	Chromosome (mouse)	exon	Amino acid
Catsper1	19A	13	686
Catsper2	2E5	16	588
Catsper3	13B1	9	395
Catsper4	4D3	12	442
Catsperβ	12E	27	1109
Catsperγ	7B1	30	1145
Catsperδ	17D	28	805

## Catsper regulation and [Ca^2+^] signaling

Two Ca^2+^ channels regulate male fertility: (1) the Orail channel, which regulate store-operated calcium entry [18], and (2) the Catsper channel, which is the most extensively studied Ca^2+^ channels in mammalian sperm [8, 11]. The sperm-specific Catsper channel controls the intracellular Ca^2+^ concentration ([Ca^2+^]i). Catsper1 and Catsper2 null mice exhibit lower amplitudes of flagellar bends compared to wild-type mice. In Catsper1 and Catsper2 null mice, the flagellar bend and amplitude are increased from abnormally low levels to normal pre-hyperactivated levels by increasing the spermospore [Ca^2+^]i [1]. In most mammals, sperm hyperactivated motility depends on calcium influx into the sperm cytoplasm either from the extracellular space or released from intracellular organelles [3, 4]. Therefore, the Catsper channel controls, at least, the swimming behavior of sperm.

### pH regulates the Catsper ion channel

**Fig. 1:**
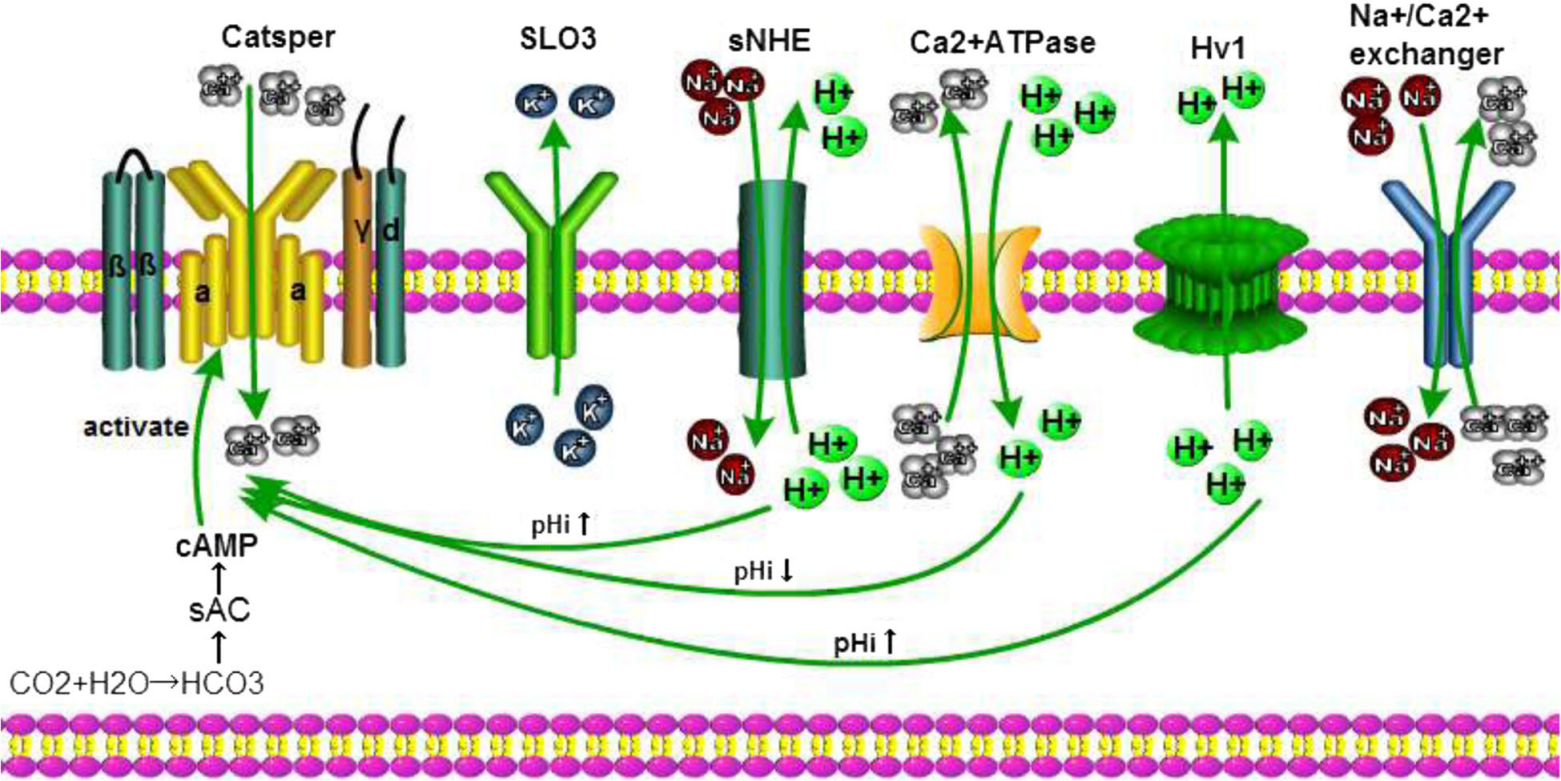
Regulation of the Catsper channel: Sperm specific K^+^ channel (SLO_3_) maintains flagellar membrane potential. Sperm Na^+^/H^+^ exchangers (NHEs) through cyclic adenosine monophosphate (cAMP) play a role on sperm fertility, while cAMP is generated in the process that bicarbonate (HCO3) activates atypical soluble adenylate cyclase (sAC). The Catsper channel is triggered by increasing intracellular PHi, which depends on sNHE and Voltage-gated H+ channel 1 (Hv1) channel pumping H^+^ out of sperm. Calcium balance in the sperm is maintained by Na^+^/Ca^2+^ exchanger and Ca^2+^ATPase exchanger. The Na^+^/Ca^2+^ exchanger exports one Ca^2+^ ion out of sperm and allows the entry of three Na^+^ ion, however, Ca^2+^ATPase is a Ca^2+^/H^+^ exchanger that removes intracellular Ca^2+^ and permits H^+^ entry into the sperm cell. Both sNHE and Hv1 channels are positive regulator of the Catsper channel, while Ca^2+^ATPase is a negative regulator of the Catsper channel

The Catsper channel is a pH-sensitive ion channel, and a high pH level is necessary for sperm hyperactivation [19]. Thus, factors that regulate the acid-base properties also affect the degree of Catsper channel-opening in sperm. According to previous work, mouse sperm produces a Ca^2+^ increase in an artificially alkalinized intracellular environment [20]. Another studies also showed that progesterone, prostaglandins and ZP3 could induce capacitation and acrosome reaction in sperm by increasing [Ca^2+^]i [21, 22]. However, later paper showed that the Ca^2+^ increases in sperm triggered by these bioactive molecules during acrosome reaction and capacitation are influenced by pH microenvironment in sperm [21]. Nevertheless, it is not completely clear how alkalinization influences the Catsper channel in sperm. H^+^ is the major regulator of acid-base microenvironment, while Na^+^/H^+^ exchangers (NHEs) and Voltage-gated H^+^ channel1 (HV1) are H^+^-relative channels. NHEs import Na^+^ into the plasma membrane and export H^+^ out of the spermatozoa while HV1 removes intracellular H^+^ to maintain the pH value (pHi) balance in spermatozoa [23]. In addition, Ca^2+^ adenosine triphosphatase (Ca^2+^ATPase) pumps remove intracellular Ca^2+^ from spermatozoa while allowing H^+^ to enter through the plasma membrane. Correspondently, the Catsper channel imports Ca^2+^ into spermatozoa to maintain Ca^2+^ homeostasis [24, 25]. Beyond that, the Na, K-ATPase (NKA) and Na^+^/Ca^2+^ exchanger (NCX) also influence the ion milieu in human sperm [26]. In fact, high levels of intracellular Ca^2+^ and low levels of intracellular H^+^ contribute to sperm hyperactivation. The relation of different ion channels with the Catsper channel is described in Fig. 1.

#### H^+^ channels

**(1) Na** ^**+**^ **/H** ^**+**^ **exchangers** Na^+^/H^+^ exchangers (NHEs) are responsible for the exchange of Na^+^ and H^+^. Also they are integral membrane proteins that are widely distributed in all prokaryotic and eukaryotic organisms. NHEs includes 13 NHE isoforms encoded by SLC9 gene family, but only three subtypes (NHE1, NHE5 and sNHE) exist in sperm cells. Among them, sNHE is a crucial isoform for fertility [27]. Namely, knockouts of the NHE1 and NHE5 genes fail to generate infertility in mice, but sNHE-null or sNHE-disruption mice became infertile due to the loss of mobility and motility of spermatozoa [3]. Increased pH levels by providing ammonium chloride recovers partial fertility, but a cAMP analogue completely recoveres fertility [28]. Furthermore, sNHE includes a nucleotide-binding domain close to the protein’s C-terminus [29]. Thus, sNHE isoform is necessary for fertility, and cyclic nucleotides may control the Catsper channel and increase pHi by activating sNHE. Moreover, sNHE helps maintain the alkaline environment, which allows the Catsper channel to further regulate hyperpolarization. Aside from maintaining the pH dynamic equilibrium in sperm, sNHE also regulates sperm maturity and promotes the absorption of salt and water in epithelial cell [27, 30]. Interfering with the function of sNHE leads to infertility. Hence, targeting sNHE may be a promising strategy for developing novel contraception methods. Like the Catsper channel, sNHE is located in the principal piece of the sperm flagellum [28], suggesting that the Catsper channel can perceive pHi changes from sNHE to regulate the Catsper function.

**(2) Hv1 channel** Hv1 is another voltage-gated H^+^ channel. Similar to sNHE and the Catsper channel, Hv1 is also located in the principal piece of sperm flagella [31]. In terms of their functions, the major difference between Hv1 and sNHE is that Hv1 maintains intracellular alkalinization only by removing intracellular H^+^ from sperm [23]. Patch-clamp techniques have detected a negative current from the Hv1 channel during the physiological process of human sperm capacitation [32], which suggests that Hv1 is associated with sperm capacitation. Interestingly, this negative current is not detected from the mouse Hv1 channel [32]. In other words, there is no H^+^ extrusion through Hv1 in mouse sperm. We suspect that sNHE independently accomplishes acid-base regulation in mouse sperm. If so, knocking out the Hv1 gene in mouse should not affect fertility, but insufficient evidence has tested this hypothesis, and the actual mechanism of Hv1 in ouse needs further investigation.

#### K^+^ channels

As mentioned previously, sNHE-induced exchange of Na^+^ and H^+^ causes pHi increases, and both sNHE and Catsper are voltage-dependent channels. In addition, cell membrane hyperpolarization correlates with capacitation. K^+^ helps maintain the balance of the membrane potential in sperm. The K^+^ channels that accomplish this balance are the SLO3 and Kir channels [33]. Among them, the SLO3 channel is a sperm-specific and pH-sensitive K^+^ channel. Similar to the Catsper channel, SLO_3_ is also strongly related to sperm hyperactivity and motility [34, 35]. Another crucial function of the SLO_3_ channel that affects the Catsper channel is to maintain the current balance of flagella in the spermospore [8]. The sperm resting transmembrane potential is approximately from −35 to -45 mV, but when K^+^ moves out of the cell and initiates hyperpolarization, the sperm transmembrane potential decreases to -70 mV [36]. A series of physiological processes are then triggered, including Na^+^/H^+^ exchanger activation, sperm capacitation and sperm binding ZP3 [37].

#### Ca^2+^ channels

There are three Ca^2+^-related channels (Catsper channel, Ca^2+^ATPase and Na^+^/Ca^2+^ exchanger) found in sperm. The Catsper channel is responsible for the entrance of Ca^2+^ into the spermospore, which promotes sperm motility. Ca^2+^ATPase is a Ca^2+^/H^+^ exchanger that removes intracellular Ca^2+^ and permits H^+^ entry into the sperm cell different from the Catsper channel [24, 25]. The Ca^2+^ATPase can negatively regulate the Catsper channel and sperm fertilization. Furthermore, the Na^+^/Ca^2+^ exchanger exports one Ca^2+^ ion out of sperm and allows the entry of three Na^+^ ion, which is essential to maintain the Ca^2+^ balance of the intracellular environment [38, 39].

#### Bicarbonate (HCO_3_^−^) transporters

HCO_3_^−^ is indispensable for sperm capacitation [40], which is often considered as the beginning of early activation of sperm motility. For example, as mentioned above, mouse sperm treated with artificial alkalinization produces Ca^2+^ increasing [20]. The addition of HCO_3_^−^ shows the same effect, while also increases the beat frequency of sperm [41]. These data suggest that transport of HCO_3_^−^ affects sperm motility by increasing the sperm pHi. Furthermore, HCO_3_^−^ activates atypical soluble adenylate cyclase (sAC), which increases the cAMP levels [42], and cAMP-mediated pathways, which increase the flagella beat frequency [43, 44](cAMP-mediated pathways can activate the Catsper channel, as mentioned below). Thus, another mechanism that HCO_3_^−^ activates the Catsper channel is the promotion of Ca^2+^ increases, which may be through enhancing the generation of cAMP. In addition, CFTR is a Cl^−^ and HCO_3_^−^ transmembrane transporter that is associated with human sperm capacitation [45]. CFTR controls many transport proteins by modulating the cAMP signaling pathway [46, 47]. Remarkably, suppression of the CFTR transporter affects HCO_3_^−^-induced cAMP increases, leading to decreased PKA activity. Moreover, decreasing CFTR activity also causes decreased tyrosine phosphorylation and decreased hyperactivated motility by modulating cAMP-downstream signaling cascades [48]. HCO_3_^−^ transporters are encoded by the SLC4, SLC26 and CFTR gene families in sperm, and these transporters constitute the main families of transmembrane proteins that are associated with the regulation of pH in mammalian cells. Western blotting, immunocytochemistry, qRT-PCR and immunoprecipitation data demonstrate that SLC26A3, SLC26A6, and SLC9A3R1 are detected in the midpieces of mouse sperm flagella. These proteins interact with each other to increase pH, during capacitation and hyperpolarization [49]. On the other hand, HCO_3_^−^ and CFTR transporters are located in the midpieces of flagella, but they do not co-localize with the Catsper channel. We speculate that HCO_3_^−^ channels influence the open or closed state of the Catsper channel indirectly by affecting the acrosome reaction.

HCO_3_^−^ signals ealy activation of sperm motility. The generation of HCO_3_^−^ is accomplished by carbonic anhydrases (CAs) (CO_2_ + H_2_O HCO_3_^−^ + H^+^). Therefore, CAs are essential for sperm during fertilization. Studies show that CAs function in three ways: (1) catalyzing the production of HCO_3_^−^, (2) regulating the pHi in sperm, and (3) regulating the sperm acrosome reaction. CAII and CAIV are the core subunits that have catalytic activities, and knocking out either of them will decrease the sperm motility, speed of sperm motility and sperm beating frequency [44]. CAII is located in the principal piece of sperm where Catsper channel is also located. CAIV, on the other hand, is located within the plasma membrane of the entire sperm tail. CA inhibitors, such as ethoxyzolamide have been administered to human capacitated sperm and mouse capacitated sperm, and the results showed that the acrosome reaction increased in human capacitated sperm, but there were no increases in mouse capacitated sperm [50]. These results demonstrated that CAs exhibited different functions in human and mouse sperm. However, little is known about how CAs participate in sperm fertilization. CAs directly participate in maintaining the balance of ions during motility. For example, the flagellar beat frequency is increased when CO_2_ is administered to the spermatozoa, and this effect is suppressed by ethoxyzolamide. Compared with the CA activity in sperm from wild-type and CAIV^−/−^ mice, physiological role of CAIV is to provide sperm, with HCO_3_^−^ required for stimulating sAC [43]. In brief, CAs can affect the alkaline environment and HCO_3_^−^ concentrations in sperm. It is conceivable that CAs may affect the sperm acrosome raction by modulating the Catsper channel.

### Physiological stimuli that regulate the Catsper channel

How extracellular Ca^2+^ enters sperm is poorly understand, but several physiological stimulus associated with fertilization induce Ca^2+^ entry to increase [Ca^2+^]i through the Catsper channel. These stimuli include progesterone, cyclic nucleotides (e.g., cAMP, cGMP), ZP glycoproteins, BSA and alkaline depolarization [51]. Collectively, these elements induce a series of physiological events, including capacitation, acrosome reaction and fertilization.

#### Cyclic nucleotide-induced Ca^2+^ entry

The cAMP/PKA signaling pathway is used in mammals to regulate gene transcription. In fact, sperm capacitation is a cAMP-dependent process that up regulates the Ca^2+^ concentration and tyrosine phosphorylation levels [52]. Cyclic AMP is synthesized by many types of adenylyl cyclases (ACs) that are commonly divided into two groups: transmembrane adenylyl cyclases (tmACs) and sAC [19]. Importantly, sAC-mediated cAMP/PKA signal cascades are essential for sperm capacitation, because sAC-mutant or sAC-null mice can produce sperm, but sperm exhibit no forward motility, causing male infertility. Giving cAMP analogs to sAC-mutant or sAC-null mice completely restores the previously lost motility, but the sperm still exhibit no hyperactivity to fertilize eggs in vitro [53, 54]. Moreover, sAC not only plays a role in producting cAMP but also participates in other mechanisms involved in the fertilization process. Specifically, sAC have three roles in fertilization. First, sAC works as a HCO_3_^−^ sensor. The structural domains of sAC rearrange after being stimulating by HCO_3_^−^, and sAC is activated by increasing cAMP levels in sperm [54, 55]. The second function of sAC is to act as a pH sensor. Previous work showed that sAC regulate acid/base equilibrium in dogfish sperm [56], and its gene works as a monitor which reflects the concentration of CO_2_ and HCO_3_^−^ to keep an appropriate pH microenvironment [55]. The last function of sAC is to function as a Ca^2+^ sensor or calmodulin. As a substitute for HCO_3_^−^, Ca^2+^ can stimulate a combination of sAC with ATP to produce cAMP [57, 58]. Cyclic AMP is a second messenger molecule that is integral to many physiological processes, including sperm chemotaxis towards to the egg and capacitation. A study showed that extracellular cAMP/cGMP increases the Ca^2+^ concentration [59]. When providing 8-Br-cAMP/cGMP to the spermatophore or providing alkaline depolarization to activate the spermatophore, a Catsper-dependent increase in the intracellular Ca^2+^ concentration initiates at the principal piece and speads through the midpiece to finally reach the head. This process occurs in a matter of seconds. Furthermore, compared with wild-type sperm, Catsper1-mutant sperm have lower intracellular ATP levels [59]. In addition, cGMP signalling function in marine invertebrates to transducer chemoattractants to increase in the [Ca^2+^]i in the flagellum, thereby increases swimming behavior during chemotaxis [60, 61]. All of these findings suggest that cyclic nucleotides induce Ca^2+^ influx in the principal piece, but there is no clear evidence showing that this cyclic nucleotide-mediated process directly participates in inducing [Ca^2+^]i increases. However, by using cells treated with 8-Br-cNMP, one study has demonstrated that a cyclic nucleotides modulates progesterone to ultimately increase [Ca^2+^]i [62].

#### ZP-induced Ca^2+^entry

An oocytes in the female reproductive tract is coated with a protective exterior called ZP. The ZP protein surrounding the oocyte is important in the fertilization process, because only when sperm pass through the ZP protein to complete the acrosome reaction can participate in fertilization. In fact, ZP glycoproteins consist of three subunits in mice: ZP1, ZP2 and ZP3 [63, 64]. Whereas four subunits-ZP1, ZP2, ZP3 and ZP4 exist in human sperm [65]. How does contact between the sperm’s acrosome and the egg’s ZP cause an increase in [Ca^2+^]i?In fact, the sperm needs capacity (which is achieved by [Ca^2+^]i increases) to pass through the ZP when the sperm contacts the egg. Exocytosis of secretory vesicles from the acrosome occur and ultimately, the acrosome reaction is accomplished [66–68]. Early research reported that in vivo experimental addition of ZP to capacitated sperm increases [Ca^2+^]i, while addition of a ZP inhibitor (i.e., tyrphostin A48, pertussis toxin and 3-quinuclidinyl benzilate) suppresses ZP-induced acrosome reactions and reduces the intracellular concentration of Ca^2+^ in mouse sperm [69–71]. These effects may be related to signal transduction through G-proteins [72]. Beta1, 4 galactosyltransferase-I (GalT-I) is a receptor of ZP3 that forms a complex of heterotrimeric G proteins. Sperm are still able to bind ZP3 in GalT-I-deleted mice, but sperm lose the ability to complete the acrosome reaction and cross the ZP [73]. Compared with sperm in mice that can complete the acrosome reaction, sperm in human not only require ZP3 binding but also require ZP4 binding [65]. In addition, mammalian transient receptor potential (Trp) proteins are Ca^2+^ channel receptors that are essential for regulating the entrance of Ca^2+^ into mouse sperm. Trp is activated by ZP3 activating G-protein and phospholipase C [74]. ZP-induced Ca^2+^ entry into sperm help generate the acrosome reaction and change sperm motility, and both of these functions are carried out with the help of Beta-defensin proteins expressed in the epididymis [75].

In recent years, studies have found that the Catsper channel plays a critical role in ZP-induced Ca^2+^ entry into mouse sperm. Using patch-clamp techniques, Ca^2+^ currents after 2 min of ZP stimulation were undetectable in Catsper1-null mouse sperm, showing that the Catsper channel is necessary for ZP to induce [Ca^2+^]i increases and suggesting that the ZP-induced [Ca^2+^]i increases start from sperm tails and propagate toward the sperm heads [64].

#### Progesterone induces Ca^2+^ increases

Progesterone surrounds the egg in the female reproductive tract and is released by cumulus cells. Progesterone induces Ca^2+^ entry into sperm through the Catsper channel and thus promotes the acrosome reaction [76, 77]. How specifically progesterone regulates Ca^2+^ concentration has been deeply investigated. An increase of the [Ca^2+^]i was discovered when human spermatozoa were exposed to a concentration gradient of progesterone to simulate sperm to approach eggs. Direct addition of Ca^2+^ to medium failed to induce this process, but increases in the [Ca^2+^]i were blocked by adding a sarcoplasmic/endoplasmic inhibitor. These data suggest that progesterone-induced Ca^2+^ influx is mediated by the release of stored Ca^2+^ in sperm, and thus may influence sperm behavior [78]. One mechanism of the Catsper channel to increase the [Ca^2+^]i is through releasing stored Ca^2+^, and we hypothesize that progesterone-induced Ca^2+^ influx may be mediated by the Catsper channel. One study recorded the Catsper currents from human epididymal and testicular spermatozoa, and the results showed that the Catsper channel is sensitive to progesterone early in sperm development and this sensitivity increases gradually to a peak when spermatozoa are ultimately ejaculated [79]. Protein kinases and phosphatases participate in progesterone-induced Ca^2+^ increases: the addition of PKA inhibitor or protein tyrosine phosphatase inhibitors reduced progesterone-induced Ca^2+^ influx and progesterone-induced acrosome rections [77]. In 2010, two research groups proposed that as a progesterone receptor in fish sperm, the Catsper channel is functioning to increase intracellular Ca^2+^ concentrations [80, 81]. In addition, in 2011, a study showed that progesterone is a steroid hormone that activates the Catsper channel in human sperm by regulating Catsper gene expression through a well-characterized progesterone nuclear receptor. Additionally, the Catsper protein is a non-genomic progesterone receptor [82], and Ca^2+^ influx is stimulated by alkaline pH and progesterone, but blocked by the Catsper inhibitors NNC55–0396 and mibefradil [83].

#### Other stimuli induce Ca^2+^ entry through the Catsper channel

One additional stimuli that promotes Ca^2+^ entry into sperm via the Catsper channel is BSA. BSA plays a role in sperm capacitation in several mammals. BSA also induces an increase in the intracellular Ca^2+^ concentration, but this effect is absent in Catsper1-knockout sperm. Addition to a EGFP-Catsper1 fusion protein recovered BSA-induced intracellular Ca^2+^ concentration increases [84]. The changes in calcium concentrations observed with BSA propagated from the principal piece to the mid-piece and, ultimately, the head within a few seconds [59].

## Functions of the Catsper protein channel

**Fig. 2:**
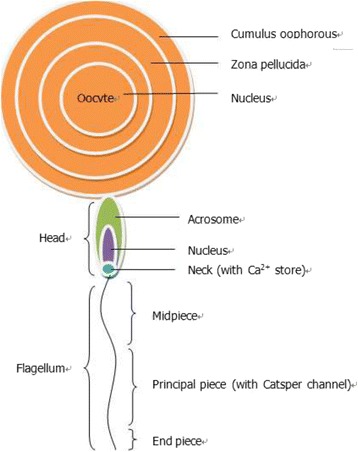
The structure of sperm and oocyte: The sperm is constituted of acrosome, nucleus, neck and flagellum. The oocyte is constituted of cumulus oophorous, ZP and nucleus. Acrosome reaction is the start of fertilization

Catsper was identified in mouse sperm as a putative Ca^2+^ channel in 2001 [9]. The Catsper channel is essential for male fertilization, especially for some physical processes, such as sperm hypermotility, egg penetration and the acrosome reaction. As such, the Catsper channel may be a target for male contraception. The structure of sperm and oocyte are showed in Fig. 2.

### Catsper and male fertility

A plethora of research has proven that the Catsper channel is essential for both human and mouse fertility. A routine semen analysis in two consanguineous families that showed autosomal-recessive male infertility discovered that both families suffer from Catsper1 gene abnormalities. In different patients, asthenoteratozoospermia was diagnosed and found to lack the Catsper2 gene [85, 86]. Furthermore, studies have also shown that all four Catsper subunits (Catsper1–4) are integral for sperm hyperactivated motility and male fertility, but lack of Catsper3 or Catsper4 has not influence spermatogenesis or initial motility of sperm [7]. Moreover, the number of sperm with progressive motility and progesterone-induced acrosome reactions is significantly lower in Catsper1-suppressived groups than that in normal groups [11]. Normal expression of the Catsper channel is associated with progressive motility and acrosome reaction, abnormal channel expression may be involved in the pathogenesis of asthenozoospermia. Specifically, disruption of the sNHE or Catsper2 genes in mice cause male infertility, with findings of immotile spermatozoa and failed hyperactivated motility, but no other apparent abnormalities [3]. All of these results suggest that Catsper1 and Catsper2 are essential for normal male fertility in humans or mice. Interestingly, Catsper3 and 4 play an important role in the acrosome reaction and male fertility [87], which occurs not only in the testes but also in other tissues. On the other hand, Catsper1 and Catsper2 are only detected in mammalian testes. Collectively these results suggest that Catsper1 and Catsper2 are highly specialized flagellar proteins that are more important than Catsper3 and Catsper4 in sperm. There is no report showing that mutations of Catpserβ or Catsperγ lead to infertility [88]. However, these mutations show an abnormal detection of Ca^2+^ currents and hyperactivated motility in spermatozoa. Finally, Catsperδ knockout mice are infertile [89].

### Catsper and contraception

The Catsper channel is a polymodal chemosensor that may be a target for contraception. Li aimed to explore the contraceptive potential of the Catsper1 transmembrane domains and pore region in vitro in human and mouse sperm. A significant decrease in sperm progressive motility was noticed after incubating cells with anti-Catsper1 lgG [90], demonstrating that Catsper1 may be a potential target for immunocontraception and that the antibody is a useful tool to study the function of ion channels in sperm. Later, Li additionally evaluated the contraceptive abilities of two B-cell epitopes in the transmembrane domains and pore region of Catsper1 in mice. Two predicted B-cell epitopes of the extracellular part of the transmembrane domains and pore region of Catsper1 were synthesized to immunize male mice. A significant reduction of fertility was observed in mating trials, with no evident systemic illnesses or abnormal mating behaviors suggesting that Catsper members may be effective and viable targets for immunocontraception. These two epitopes in Catsper1 share high identity between mouse and human and thus may be effective for regulating fertility in humans [91]. Novel drugs targeting the Catsper channel are warranted to study their potential roles in reversibly acting as male contraception.

## Pharmacological targeting of the Catsper channel

With more advanced emerging methods that employ animal or cell models, more studies testing drugs that act as Catsper agonists or inhibitors are needed to test the channel’s potential role as a target for infertility treatment and male contraception. The drugs that target the Catsper channel can be divided into three groups according to their pharmacological actions. Few of them are approved by the US Food and Drug Administration (FDA) for clinical therapies. Most of these drugs are at the stage of laboratory experiments, while a large portion of them are plant extracts.

### Anticholinesterase drugs

In early studies, the organochlorine compounds of semen in infertile men have been analyzed, and the results showed that the concentrations of dichlorodiphenyltrichloroethane (DDT) and its metabolites, such as hexachlorocyclohexane (HCH), P,P′-dichlorodiphenyldichloroethylene (pp’-DDE) and pp’-DDD, are higher in the semen of infertile men than that in fertile men. It has been demonstrated that anticholinesterases affect male fertility by damaging the prostate [92–95]. Another in-vitro experiment, however, found that P,P′-DDE was a Catsper agonist that stimulated Catsper channel opening and caused Ca^2+^ influx into sperm [96]. In other words, P,P′-DDE may improve sperm fertility. Thus, P,P,-DDE is a controversial compound compounds in sperm fertility.

### Ca^2+^ channel blockers

Compounds such as HC-056456, NNC55–0396, nifedipine, nimodipine, quinindium, clofilium, theophylline and ketamine are all Ca^2+^ channel blockers. Among them, HC-056456 is a novel Ca^2+^ channel blocker, which is reported to be a unique compound that selectively targets the Catsper channel. Whole cell patch-clamp recordings showed a lower Catsper current in HC-056456 treated sperm than in untreated sperm, and HC-056456 reversibly prevented the development of hyperactivated motility of capacitated sperm [97]. This effect is similar to the findings of Catsper-null sperm, thus, HC-056456 is a promising compound that should be studied further as a male contraceptive. Both nifedipine and nimodipine are L-type Ca^2+^ channel blockers [98, 99], and 20 mg/L of both compounds could induce male infertility. Nifedipine also targets the Catsper chanel and prevents Ca^2+^ influx into sperm, which consequently alters the cholestenone content in sperm membranes leading to membrane disruption [10]. NNC55–0396 and mibefradil are two T-type Ca^2+^ channel blockers that are odorants and suppress Ca^2+^ signals under standard (physiological) conditions [100]. NNC (10 mM) and Mib (30 mM) significantly decrease the percentage of sperm with progressive motility and other kinematic parameters, but the compounds do not affect the percentage of hyperactivated sperm [11]. Other odorants, such as cyclamen and helional, evoke Ca^2+^ signals [51], these compounds are extracted from plants and bacteria and may act as potent molecules to treat Catsper-related male infertility. In addition, whole cell patch-clamp recordings of human sperm Catsper ion channels showed that quinidine reversibly blocks Ca^2+^ currents in the Catsper channel. Clofilium, on the other hand, causes irreversible blockade of Catsper-mediated Ca^2+^ currents [100–102]. A recent study showed that ketamine affects human sperm functions as well by inhibiting sperm total motility and progressive motility via decreased sperm Ca^2+^ influx [103].

### Other therapeutics that affect the Catsper channel

The mechanisms of some drugs are not clearly understand. For example, emodin inhibits human sperm function by reducing the sperm [Ca^2+^]i and tyrosine phosphorylation [104]. Furthermore, some herbal plants, such as Trigonellae Semen (TS) and *Panax ginseng*, generate hyperactivity of sperm by regulating the expression of the Catsper gene. Extracting pure compounds from both TS and *Panax ginseng* may cure oligoasthenospermia [105, 106]. In addition, matrine significantly inhibits sperm total motility, capacitation linear velocity and the progesterone-induced acrosome reaction by stimulating the Catsper channel [107], thus, matrine could be an potent drug to treat male contraception, While further clinic trails and systematic evaluations of these molecules are warranted.

At this point, drugs targeting on the Catsper channel remains in preclinical research stages. More intensive study of the Catsper channel as a target for treating is needed.

## The effects of the Catsper gene promoter on its transcription

The regulation of the Catsper channel has been systematically studied at the protein level, but few researches reveal the effects of the Catsper promoter regions on the transcription of the Catsper gene. One group studied the promoter regions of the Catsper channel in human and murine sperm. Electrophoretic Mobility Shift Assays (EMSA) and DNA footprinting techniques were used to analyze the Catsper gene characteristics. There is a retardant when the Catsper gene and nucleoprotein were incubated together in vitro, suggesting some nucleoproteins have combined with Catsper gene promoter. Moreover, three transcription factor binding sites for SRY, SOX and CREB have been found in the Catsper promoter regions [108]. This group also demonstrated that the transcription factors SOX5 and SOX9 regulate the expression of the Catsper1 gene [109]. The role of the other transcription factors are still unknown. In addition, a toxicology study showed that cyclophosphamide (CP), a antineoplastic drug, could cause male infertility and lead to a significant reduction of the CREM transcription factor within the CREB transcription factor family [110]. The concentrations of CREM in spermatophores is 100 times greater than in other organizations of cells [111]. CREM-knockout mice also induce infertility. Thus, CREM is an important transcription factor in regulating the Catsper channel.

## Conclusion and perspective

The Catsper channel plays a critical role in male fertility by controlling Ca^2+^ influx into spermatozoa. Many studies revealed protein and hormone based regulatory mechanism of the Catsper channel. In this review, we discussed how ion channels and stimulants influence the Catsper channel and induce Ca^2+^ entry into sperm. Thus far, a variety of Ca^2+^ channels have been found, and these channels (i.e., high voltage-gated Ca^2+^ channel (Cav), cyclic nucleotide-gated Ca^2+^ channel (CNG) and the TRP channel) are distributed in different subregions of sperm. In addition, pharmacological evidence shows that there are N-type, R-type and T-type voltage-gated Ca^2+^ channels in sperm cells, but these channels do not directly affect sperm movement and fertility [112]. Only the Catsper channel directly modulates the physiological processes of sperm hyperactivation, sperm capacitation, chemotaxis towards to the egg and the acrosome reaction [8]. Many questions remain unanswered, though. Catsper is a pH-sensitive ion channel. Some ion channels or enzymes (i.e., sNHE, CAs and HCO_3_^−^ transporter) alter sperm pH by changing the concentration of H^+^ ions. We know that all these biological molecules affect the opening degree of the Catsper channel, but are all of them essential for the Catsper channel? If one of them is mutated or deleted, can the Catsper channel continue to function normally? The mechanism of how these biological molecules interact with each other is not very clear. Furthermore, the sperm sNHE exchanger also acts via cAMP, however, the mechanism has not been clearly demonstrated. On the other hand, cAMP, ZP, progesterone and BSA are proteins that promote Ca^2+^ entry into Catsper channel. These compounds promote capacitation, the acrosome reaction, sperm maturation and sperm combining with an egg. What prevents activation of Catsper1 and Catsper2 in heterologous systems, and what other cell conditions are required to achieve activation? Other than the ZP, progesteronel, nucleotides and BSA, what other elements can increase Ca^2+^ influx through the Catsper channel? All of these questions need further exploration.

In addition, Catsper channel controls Ca^2+^ flux into spermatozoa and adjusts to hyperactivation of sperm. Thus, Catsper channel has been implicated as a potential target for contraception, and there are a lot of potent Catsper channel blockers for contraception, such as HC-056456, NNC55–0396 and so on. Moreover, in 2017, Zou also found that diethylstilbestrol (DES), a Catsper activator, facilitated Ca^2+^ flux into human spermatozoa by Catsper channel [113]. Even so, the studies on Catsper agonists is still very rare. So it is necessary to study the mechanism of Catsper channel to research Catsper-targeted drugs treating male infertility.

The Catsper channel is a unique Ca^2+^ channel that is only detected in testes, so we speculate that Catsper may have unique factors or protein-protein interactions that contribute to the unique properties and regulation of the Catsper channel. In fact, there are few studies about the channel’s promoter regions, and only four binding sequences on the Catsper1 gene promoter have been discovered [73]. No studies have described the remainder of the Catsper gene, so searching for new Catsper promoter binding sequences is important for finding potential molecular targets that could be used to treat male infertility. In addition, at the protein level, there are only two transcription factors (SOX5 and SOX9) that have been shown to regulate the transcription of the Catsper1 gene [109]. The SOX protein is a ubiquitously utilized transcription factor in many organisms, so the existence of another more specific protein that acts only on the Catsper promoter sequence may be an important area for future studies.
